# Correction: Controversy matters: Impacts of topic and solution controversy on the perceived credibility of a scientist who advocates

**DOI:** 10.1371/journal.pone.0196620

**Published:** 2018-04-24

**Authors:** Lindsey Beall, Teresa A. Myers, John E. Kotcher, Emily K. Vraga, Edward W. Maibach

The following information is missing from the Funding section: Publication of this article was funded in part by the George Mason University Libraries Open Access Publishing Fund (https://library.gmu.edu/about/oa-fund).
