# Correction: Controversy matters: Impacts of topic and solution controversy on the perceived credibility of a scientist who advocates

**DOI:** 10.1371/journal.pone.0211289

**Published:** 2019-01-18

**Authors:** Lindsey Beall, Teresa A. Myers, John E. Kotcher, Emily K. Vraga, Edward W. Maibach

After publication, the authors became aware of errors to the cleaned data set used in the analysis presented in the study. The authors re-downloaded the data from Qualtrics, re-cleaned the data, and were able to run the analyses correctly. Multiple members of the team were able to replicate the updated findings separately. The interpretation of the results and overall understanding of the study has not changed. All the relationships that were significant in the published version are significant with the updated data set; however, several coefficients in Tables 2–5 and S1 Table are incorrect. Please view the corrected Tables 2–5 and S1 Table here.

**Table 2:** Total effects of non-controversial and controversial solutions on credibility, in comparison to the information only condition.

		Credibility
Controversial vs. Information Only		
	Flu	-0.38**
	Marijuana	-0.17
	Severe Weather	0.26*
	Climate Change	-0.01
Non-Controversial vs. Information Only		
	Flu	0.22+
	Marijuana	-0.05
	Severe Weather	0.28*
	Climate Change	0.30*

Note: Entries are unstandardized regression coefficients and can be interpreted as the difference in the means between the two conditions.

+ *p* < .10,

* *p* < .05,

** *p* < .01

*** *p* < .001

**Table 3:** Effect of solution position on perceived motivations of the scientist.

	Scientific Evidence	Inform Public	Serve Public	Persuade the Public	Personal Promotion	Political Views
Non-Controversial vs. Information Only						
Flu	0.11	0.18	0.41**	0.58***	0.01	-0.12
Marijuana	0.03	0.04	0.13	0.36*	-0.21	0.04
Severe Weather	0.33*	-0.14	0.43***	1.19***	-0.01	-0.14
Climate Change	0.13	0.15	0.35*	0.60***	-0.19	0.28
Controversial vs. Information Only						
Flu	-0.12	-0.68***	-0.37**	0.50***	0.31+	0.54**
Marijuana	0.07	-0.43*	-0.15	0.31*	0.02	0.17
Severe Weather	0.20	-0.28+	0.35**	1.25***	0.11	0.21
Climate Change	-0.03	-0.17	0.03	0.37**	0.04	0.33+

Note: Entries are unstandardized regression coefficients and can be interpreted as the difference in means between the two conditions.

+ *p* < .10,

* *p* < .05,

** *p* < .01,

*** *p* < .001

**Table 4:** Perceived motivations relationships with credibility.

	Credibility			
	Flu	Marijuana	Severe weather	Climate change
Scientific evidence	0.22***	0.26***	0.31***	0.26***
Inform public	0.09***	0.18***	0.06*	0.16***
Serve public	0.34***	0.31***	0.24***	0.25***
Persuade public to take action	0.12***	0.03	0.06+	0.11***
Personal promotion/gain	-0.10***	-0.12***	-0.09***	-0.10***
Political views	-0.02	-0.04	-0.04	0.01

Note: Entries are unstandardized regression coefficients from regressions predicting credibility from the motivations; one regression was conducted for each topic.

*p < .05,

**p < .01,

***p < .001

**Table 5:** Indirect effects of solution position on credibility, through perceived scientist motivations.

	Indirect Effect on Credibility					
	Via scientific evidence	Via inform the public	Via serve the public	Via persuade the public	Via personal promotion	Via political views
Non-Controversial vs. Information Only						
Flu	0.03	0.02	0.14*	0.07*	0.00	0.00
Marijuana	0.01	0.01	0.04	0.01	0.02	0.00
Severe Weather	0.10*	-0.01	0.10*	0.07	0.00	0.01
Climate Change	0.03	0.02	0.09*	0.07*	0.02	0.00
Controversial vs. Information Only						
Flu	-0.03	-.06*	-0.13*	0.06*	-0.03	-0.01
Marijuana	0.02	-.08*	-0.04	0.01	0.00	-0.01
Severe Weather	0.06	-.02*	0.08*	0.08	-0.01	-0.01
Climate Change	-.01	-0.03	0.01	0.04*	0.00	0.00

Note: Entries are unstandardized indirect effects, generated by the PROCESS macro (Hayes, 2013). See Table 1 for Total Effects. Indirect effects of non-controversial vs. controversial solution position are available in the supplemental material.

*p < .05

[1]

## Supporting information

S1 Table Analysis moderated by political ideology. (DOCX)
