# Supplementary material for: Controversy matters: Impacts of topic and solution controversy on the perceived credibility of a scientist who advocates
Source: PLoS One. 2017 Nov 14;12(11):e0187511. doi: 10.1371/journal.pone.0187511 (PMC5685625; doi:10.1371/journal.pone.0187511)

Appendix 1A

Experimental Stimuli

Flu ­ Informative

Dr. Dave Wilson, a recognized international expert in the field of public health, recently published an op­ed in USA Today. In the article, he said:

“Evidence from recent scientific studies reveal that the flu is even more dangerous and costly than once believed. The flu contributes to pneumonia, bronchitis, fatigue, and even premature death. Moreover, since so many people are affected by the flu, it is increasing everyone's insurance costs."

Flu ­ Non-controversial

Dr. Dave Wilson, a recognized international expert in the field of public health, recently published an op­ed in USA Today. In the article, he said:

“Evidence from recent scientific studies reveal that the flu is even more dangerous and costly than once believed. The flu contributes to pneumonia, bronchitis, fatigue, and even premature death. Moreover, since so many people are affected by the flu, it is increasing everyone's insurance costs.

The flu threatens everyone and action should be urgently taken to reduce the risk. One effective approach for addressing the problem of the flu is to introduce legislation to post information signs about the link between hand washing and the flu in all restaurants and public buildings. My research, and research conducted by many other experts, suggests that this simple and inexpensive action is an effective way to protect Americans from the risks of the flu."

Flu ­ Controversial

Dr. Dave Wilson, a recognized international expert in the field of public health, recently published an op­ed in USA Today. In the article, he said:

“Evidence from recent scientific studies reveal that the flu is even more dangerous and costly than once believed. The flu contributes to pneumonia, bronchitis, fatigue, and even premature death. Moreover, since so many people are affected by the flu, it is increasing everyone's insurance costs.

The flu threatens everyone and action should be urgently taken to reduce the risk. One effective approach for addressing the problem of the flu is to introduce legislation to require all Americans to get flu shots every year. My research, and research conducted by many other experts, suggests that this simple and inexpensive action is an effective way to protect Americans from the risks of the flu."

Marijuana ­ Informative

Dr. Dave Wilson, a recognized international expert in the field of public health, recently published an op­ed in USA Today. In the article, he said:

“Evidence from recent scientific studies reveal that marijuana use is even more dangerous and costly than once believed. Marijuana use contributes to mental health problems, including depression and anxiety disorders, and it is addictive for many users. Moreover, because so many people are affected by marijuana use, it is increasing everyone’s insurance costs."

Marijuana ­ Non-controversial

Dr. Dave Wilson, a recognized international expert in the field of public health, recently published an op­ed in USA Today. In the article, he said:

“Evidence from recent scientific studies reveal that marijuana use is even more dangerous and costly than once believed. Marijuana use contributes to mental health problems, including depression and anxiety disorders, and it is addictive for many users. Moreover, because so many people are affected by marijuana use, it is increasing everyone’s insurance costs.

Marijuana use threatens everyone and action should be urgently taken to reduce the risk. One effective approach for addressing the problem of marijuana use is to introduce legislation to require warning labels placed on all commercially sold marijuana products. My research, and research conducted by many other experts, suggests that this simple and inexpensive action is an effective way to protect Americans from the risks of marijuana use."

Marijuana ­ Controversial

Dr. Dave Wilson, a recognized international expert in the field of public health, recently published an op­ed in USA Today. In the article, he said:

“Evidence from recent scientific studies reveal that marijuana use is even more dangerous and costly than once believed. Marijuana use contributes to mental health problems, including depression and anxiety disorders, and it is addictive for many users. Moreover, because so many people are affected by marijuana use, it is increasing everyone’s insurance costs.

Marijuana use threatens everyone and action should be urgently taken to reduce the risk. One effective approach for addressing the marijuana use problem is to introduce legislation to create stricter regulation on medical marijuana distribution and to prohibit city and state legalization of marijuana. My research, and research conducted by many other experts, suggests that this simple and inexpensive action is an effective way to protect Americans from the risks of marijuana use."

Severe weather ­ Informative

Dr. Dave Wilson, a recognized international expert in the field of meteorology, recently published an op­ed in USA Today. In the article, he said:

“Evidence from recent scientific studies reveals that severe weather is more dangerous and costly than once believed. Severe weather (such as floods and hurricanes) contributes to injuries, deaths and property damage, and has increased markedly in recent years. Moreover, because so many people are affected by severe weather, it is increasing everyone’s insurance costs."

Severe weather ­ Non­controversial

Dr. Dave Wilson, a recognized international expert in the field of meteorology, recently published an op­ed in USA Today. In the article, he said:

“Evidence from recent scientific studies reveals that severe weather is more dangerous and costly than once believed. Severe weather (such as floods and hurricanes) contributes to injuries, deaths and property damage, and has increased markedly in recent years. Moreover, because so many people are affected by severe weather, it is increasing everyone’s insurance costs.

Severe weather threatens everyone and action should be urgently taken to reduce the risk. One effective approach for addressing the problem of severe weather is to introduce legislation to implement better early warning systems in communities throughout the country. My research, and research conducted by many other experts, suggests that this simple and inexpensive action is an effective way to protect Americans from the risks of severe weather."

Severe weather ­ Controversial

Dr. Dave Wilson, a recognized international expert in the field of meteorology, recently published an op­ed in USA Today. In the article, he said:

“Evidence from recent scientific studies reveals that severe weather is more dangerous and costly than once believed. Severe weather (such as floods and hurricanes) contributes to injuries, deaths and property damage, and has increased markedly in recent years. Moreover, because so many people are affected by severe weather, it is increasing everyone’s insurance costs.

Severe weather threatens everyone and action should be urgently taken to reduce the risk. One effective approach for addressing the severe weather problem is to introduce legislation to restrict building and development in areas known to be at high­risk for severe weather. My research, and research conducted by many other experts, suggests that this simple and inexpensive action is an effective way to protect Americans from the risks of severe weather."

Climate change ­ Informative

Dr. Dave Wilson, a recognized international expert in the field of meteorology, recently published an op­ed in USA Today. In the article, he said:

“Evidence from recent scientific studies reveal that climate change is more dangerous and costly than once believed. Carbon dioxide contributes to damage to human health, food shortages, species loss, and economic impacts on the order of 5% of global GDP. Moreover, because so many people are affected by climate change, it is increasing everyone’s insurance costs."

Climate change ­ Non­controversial

Dr. Dave Wilson, a recognized international expert in the field of meteorology, recently published an op­ed in USA Today. In the article, he said:

“Evidence from recent scientific studies reveal that climate change is more dangerous and costly than once believed. Carbon dioxide contributes to damage to human health, food shortages, species loss, and economic impacts on the order of 5% of global GDP. Moreover, because so many people are affected by climate change, it is increasing everyone’s insurance costs.

Climate change threatens everyone and action should be urgently taken to reduce the risk. One effective approach for addressing the problem of climate change problem is to introduce legislation to provide tax rebates to people who purchase energy efficient vehicles or solar panels. My research, and research conducted by many other experts, suggests that this simple and inexpensive action is an effective way to protect Americans from the risks of climate change."

Climate change ­ Controversial

Dr. Dave Wilson, a recognized international expert in the field of earth science, recently published an op­ed in USA Today. In the article, he said:

“Evidence from recent scientific studies reveal that climate change is more dangerous and costly than once believed. Carbon dioxide contributes to damage to human health, food shortages, species loss, and economic impacts on the order of 5% of global GDP. Moreover, because so many people are affected by climate change, it is increasing everyone’s insurance costs.

Climate change threatens everyone and action should be urgently taken to reduce the risk. One effective approach for addressing the climate change problem is to introduce legislation to apply a carbon tax on all fossil fuels including coal, oil, and natural gas. My research, and research conducted by many other experts, suggests that this simple and inexpensive action is an effective way to protect Americans from the risks of climate change."

Appendix 1B

Questionnaire

The primary goal of Dr. Wilson's op­ed in USA Today was to provide impartial information about [topic]

Strongly Disagree

Disagree

Somewhat Disagree

Neither Agree nor Disagree

Somewhat Agree

Agree

Strongly Agree

The primary goal of Dr. Wilson's op­ed in USA Today was to persuade people to take action about [topic]

Strongly Disagree

Disagree

Somewhat Disagree

Neither Agree nor Disagree

Somewhat Agree

Agree

Strongly Agree

Dr. Wilson’s op-ed in USA Today was motivated by his evaluation of the scientific evidence of the issue

Strongly Disagree

Disagree

Somewhat Disagree

Neither Agree nor Disagree

Somewhat Agree

Agree

Strongly Agree

Dr. Wilson’s op-ed in USA Today was motivated by his political views on the issue

Strongly Disagree

Disagree

Somewhat Disagree

Neither Agree nor Disagree

Somewhat Agree

Agree

Strongly Agree

Dr. Wilson’s op-ed in USA Today was motivated by his desire for personal promotion and gain

Strongly Disagree

Disagree

Somewhat Disagree

Neither Agree nor Disagree

Somewhat Agree

Agree

Strongly Agree

Dr. Wilson’s op-ed in USA Today was motivated by his desire to serve the public

Strongly Disagree

Disagree

Somewhat Disagree

Neither Agree nor Disagree

Somewhat Agree

Agree

Strongly Agree

Appendix 1C

Please indicate your impression of Dr. Wilson by choosing the appropriate number between the pairs of adjectives below. The closer then number is to either adjective, the more certain you are of your evaluation.

Not at all expert 1 – 2 – 3 – 4 – 5 – 6 – 7 – 8 Extremely expert

Extremely sincere 1 – 2 – 3 – 4 – 5 – 6 – 7 – 8 Not at all sincere

Not at all sensitive 1 – 2 – 3 – 4 – 5 – 6 – 7 – 8 Extremely sensitive

Not at all competent 1 – 2 – 3 – 4 – 5 – 6 – 7 – 8 Extremely competent

Not at all trustworthy 1 – 2 – 3 – 4 – 5 – 6 – 7 – 8 Extremely trustworthy

Is concerned about society a great deal 1 – 2 – 3 – 4 – 5 – 6 – 7 – 8 Isn’t concerned about society a great deal

Not at all intelligent 1 – 2 – 3 – 4 – 5 – 6 – 7 – 8 Intelligent

Not at all honest 1 – 2 – 3 – 4 – 5 – 6 – 7 – 8 Extremely honest

Note: Although this is a 7-point scale, participants were able to select one of eight radio buttons. We have acknowledged this issue in the limitations section of the main document.


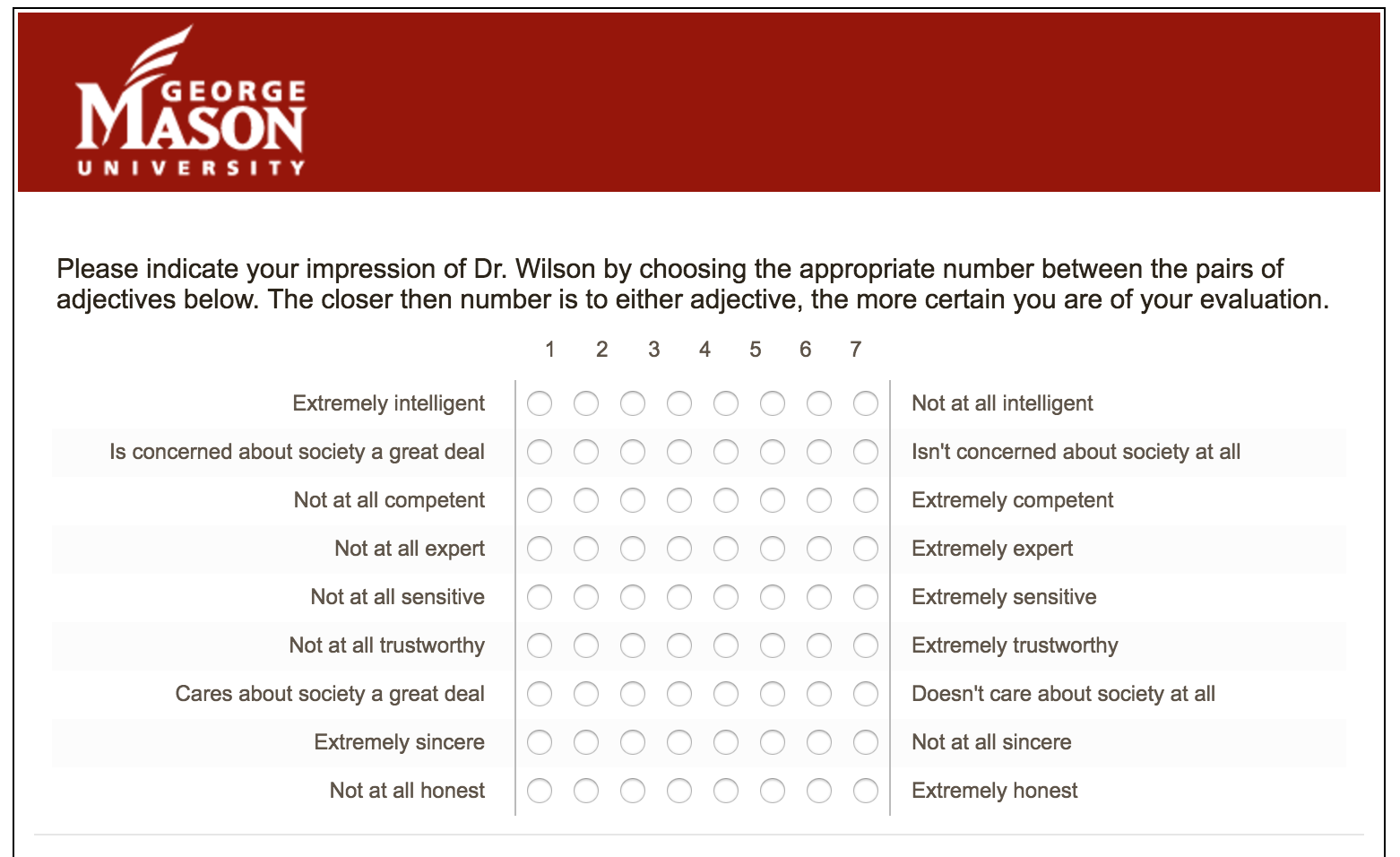

Supplement: S1 Appendix — (DOCX) [file pone.0187511.s001.docx]
