# Supplementary material for: Correction: Controversy matters: Impacts of topic and solution controversy on the perceived credibility of a scientist who advocates
Source: PLoS One. 2019 Jan 18;14(1):e0211289. doi: 10.1371/journal.pone.0211289 (PMC6338381; doi:10.1371/journal.pone.0211289)
Supplement: S1 Table — (DOCX) [file pone.0211289.s001.docx]

S1 Tables

In response to a request from the reviewers of this manuscript, we tested whether there was an interaction between political ideology and any of the links shown in this paper, as a way to determine whether different results across topics may be due to differences in how people of various political ideologies responded to that specific topic. We used the PROCESS macro to examine political ideology as a moderating variable (at the end of the survey, participants were asked to report their political ideology, which ranged from very liberal (coded 1) to very conservative (coded 5), *M* = 3.05, *SD* = 1.07). Results are shown here.

Table S1A. Moderated Effects of Non-controversial and Controversial Solutions on Credibility, in Comparison to the Information Only Condition

|  |  | Credibility | | | |
| --- | --- | --- | --- | --- | --- |
|  |  | Solution position x Ideology (omnibus interaction) | Specific Effects | | |
| Non-controversial vs. Information Only | |  | Liberal | Moderate | Conservative |
| Flu |  | -.15 | -- | -- | -- |
| Marijuana |  | .17 | -- | -- | -- |
| Severe Weather |  | .00 | -- | -- | -- |
| Climate Change |  | .01 | -- | -- | -- |
| Controversial vs. Information Only | |  |  |  |  |
| Flu |  | -.09 | -- | -- | -- |
| Marijuana |  | .30* | -.53* | -- | -- |
| Severe Weather |  | -.03 | -- | -- | -- |
| Climate Change |  | .04 | -- | -- | -- |

*** p < .001; ** p < .01; * p < .05

|  | | Omnibus Interaction of Political Ideology by: | | | | | |
| --- | --- | --- | --- | --- | --- | --- | --- |
|  | | Scientific Evidence | Inform Public | Serve Public | Persuade the Public | Personal Promotion | Political Views |
| Non-controversial vs. Information Only | |  |  |  |  |  |  |
|  | Flu | -.10 | -.03 | -.08 | -.06 | .07 | -.06 |
|  | Marijuana | .05 | .07 | .16 | .04 | .08 | -.12 |
|  | Severe Weather | -.11 | .18 | .20+ | -.05 | -.31+ | -.08 |
|  | Climate change | -.08 | .14 | .11 | .02 | -.05 | -.14 |
| Controversial vs. Information Only | |  |  |  |  |  |  |
|  | Flu | -.09 | -.18 | -.19+ | -.04 | -.13 | -.31+ |
|  | Marijuana | .06 | .22 | .19 | .05 | .04 | -.11 |
|  | Severe Weather | -.13 | -.28+ | .18 | -.15 | -.24 | -.08 |
|  | Climate change | -.08 | -.02 | .00 | .07 | -.14 | -.11 |

Table S1B. Non-controversial and Controversial Solutions on Perceived Motivations of the Scientist Moderated by Political Ideology, Omnibus Interactions

Note: No omnibus interactions were significant, therefore, the specific effects are not included.

*** p < .001; ** p < .01; * p < .05

Table S1C. The Relationship between Perceived Motivations and Credibility Moderated by Political Ideology

|  | Flu | | | | | | | Marijuana | | | | |  |
| --- | --- | --- | --- | --- | --- | --- | --- | --- | --- | --- | --- | --- | --- |
|  | Motivation x Ideology (omnibus interaction) | Specific Effects | | | | | | Motivation x Ideology (omnibus interaction) | Specific Effects | | | |  |
|  |  | Lib. | Mod. | | | Cons. | |  | Lib. | Mod. | Cons. | |  |
| Scientific Evidence | .11*** | .43*** | .55*** | | | .66*** | | -.01 | -- | -- | -- | |  |
| Inform Public | .06* | .21*** | .28*** | | | .34*** | | -.06** | .47*** | .41*** | .34*** | |  |
| Serve Public | .10*** | .52*** | .63*** | | | .73*** | | -.04 | -- | -- | -- | |  |
| Persuade Public | .04 | -- | -- | | | -- | | .04 | -- | -- | -- | |  |
| Personal Promotion | -.04 | -.10** | -.15*** | | | -.20*** | | -.08** | - .19*** | -.27*** | -.36*** | |  |
| Political Views | .02 | -- | -- | | | -- | | -.03 | -- | -- | -- | |  |
|  |  |  |  | | |  | |  |  |  |  | |  |
|  | Severe Weather | | | | | | | Climate Change | | | | | |
|  | Motivation x Ideology (omnibus interaction) | Specific Effects | | | | | | Motivation x Ideology (omnibus interaction) | Specific Effects | | | | |
|  |  | Lib. | | Mod. | | | Cons. |  | Lib. | Mod. | | Cons. | |
| Scientific Evidence | .03 | -- | | -- | | | -- | .04 | -- | -- | | -- | |
| Inform Public | .07** | .13** | | .20*** | | | .28*** | .05* | .24*** | .30*** | | .35*** | |
| Serve Public | .02 | -- | | -- | | | -- | .03 | -- | -- | | -- | |
| Persuade Public | .05+ | -.15*** | | | -.20*** | | -.25*** | .02 | -- | -- | | -- | |
| Personal Promotion | -.03 | -- | | -- | | | -- | -.08** | -.08* | -.17*** | | -.25*** | |
| Political Views | -.03 | -- | | -- | | | -- | -.03 | -- | -- | | -- | |

*** p < .001; ** p < .01; * p < .05
